# PI3-kinase deletion promotes myelodysplasia by dysregulating autophagy in hematopoietic stem cells

**DOI:** 10.1126/sciadv.ade8222

**Published:** 2023-02-22

**Authors:** Kristina Ames, Imit Kaur, Yang Shi, Meng M. Tong, Taneisha Sinclair, Shayda Hemmati, Shira G. Glushakow-Smith, Ellen Tein, Lindsay Gurska, Ulrich Steidl, Robert Dubin, Jidong Shan, Cristina Montagna, Kith Pradhan, Amit Verma, Kira Gritsman

**Affiliations:** ^1^ Department of Medicine, Albert Einstein College of Medicine, Bronx, NY, USA.; ^2^ Department of Cell Biology, Albert Einstein College of Medicine, Bronx, NY, USA.; ^3^ Department of Pathology, Montefiore Hospital, Bronx, NY, USA.; ^4^ Department of Genetics, Albert Einstein College of Medicine, Bronx, NY, USA.; ^5^ Department of Radiation Oncology and Genomic Instability and Cancer Genetics, Rutgers Cancer Institute of New Jersey, NJ, USA.; ^6^ Department of Medical Oncology, Montefiore Hospital, Bronx, NY, USA.; ^7^ Department of Developmental and Molecular Biology, Albert Einstein College of Medicine, Bronx, NY, USA.

## Abstract

Myelodysplastic syndrome (MDS) is a clonal malignancy arising in hematopoietic stem cells (HSCs). The mechanisms of MDS initiation in HSCs are still poorly understood. The phosphatidylinositol 3-kinase (PI3K)/AKT pathway is frequently activated in acute myeloid leukemia, but in MDS, PI3K/AKT is often down-regulated. To determine whether PI3K down-regulation can perturb HSC function, we generated a triple knockout (TKO) mouse model with *Pik3ca*, *Pik3cb*, and *Pik3cd* deletion in hematopoietic cells. Unexpectedly, PI3K deficiency caused cytopenias, decreased survival, and multilineage dysplasia with chromosomal abnormalities, consistent with MDS initiation. TKO HSCs exhibit impaired autophagy, and pharmacologic autophagy induction improved HSC differentiation. Using intracellular LC3 and P62 flow cytometry and transmission electron microscopy, we also observed abnormal autophagic degradation in patient MDS HSCs. Therefore, we have uncovered an important protective role for PI3K in maintaining autophagic flux in HSCs to preserve the balance between self-renewal and differentiation and to prevent MDS initiation.

## Teaser

PI3 kinase maintains recycling of cellular components to preserve bone marrow stem cell function and prevent MDS initiation.

## INTRODUCTION

Myelodysplastic syndrome (MDS) is characterized by the clonal expansion of dysfunctional hematopoietic stem cells (HSCs) with molecular and/or cytogenetic abnormalities, leading to impaired myeloid and erythroid differentiation, which results in multilineage cytopenias. Patients with MDS often suffer from transfusion dependence, an increased risk of infection and bleeding, and variable progression to acute myeloid leukemia (AML), all of which are associated with substantial mortality (*1*). While the cytogenetic and molecular events in MDS are well delineated, the mechanisms that promote the initiation of dysplasia in HSCs and lead to impaired myeloid and erythroid differentiation are still poorly understood.

Many growth factors, cytokines, and chemokines that contribute to maintaining the intricate balance between self-renewal and differentiation in HSCs signal through the phosphatidylinositol 3-kinase (PI3K)/protein kinase B (AKT) pathway. Class PI3Ks are heterodimeric lipid kinases, and in mammals, all three class IA p110 catalytic isoforms (P110α, P110β, and P110δ), encoded by *PIK3CA, PIK3CB*, and *PIK3CD*, respectively, bind to the same regulatory subunits and can functionally compensate for one another (*2*). The class I PI3Ks produce the lipid second messenger phosphatidylinositol (3,4,5)-trisphosphate (PIP_3_), which aids in recruitment of the serine/threonine (Ser/Thr) kinase AKT from the cytosol to the plasma membrane, where it becomes activated, leading to activation of its downstream effectors, such as the mechanistic target of rapamycin (MTOR) and its substrates S6 kinase and eukaryotic translation initiation factor 4E-binding protein 1 (4EBP1) (*3*). To limit AKT activation, PIP_3_ production can be antagonized by three phosphatases: phosphatase and tensin homolog (PTEN), Src homology 2 (SH2) domain containing inositol polyphosphate 5-phosphatase 1 (SHIP1), and inositol polyphosphate 4-phosphatase type II (INPP4B) (*2*, *4*). Hyperactivation of PI3K/AKT in mouse hematopoietic cells, either directly via AKT activation or indirectly via PTEN deficiency, is deleterious for normal HSC function, causing increased cycling, with consequent depletion of the stem cell pool (*5*–*9*).

Pathologic phosphorylation of AKT has been reported in up to 80% of AML cases (*10*). However, AKT activation is much less frequently observed in patients with MDS and seen in only 5% of high-risk MDS cases as reported by Nyåkern *et al.* (*11*). One RNA sequencing study in MDS patient CD34 cells compared to healthy donor CD34 cells revealed down-regulation of a PI3K signaling expression signature and up-regulation of a PTEN signature (*12*). Given the importance of the PI3K/AKT pathway in HSC homeostasis and the unclear role of this pathway in MDS, we sought to investigate the roles of the PI3K/AKT pathway in MDS initiation and progression.

In a larger MDS RNA sequencing dataset (*13*), we observed an increase in the expression of multiple phosphatases, including *PTEN, INPP4B*, and *SHIP1*, in MDS patient CD34^+^ cells, compared to healthy CD34^+^ cells, which would be predicted to decrease PI3K/AKT signaling in MDS stem cells. Therefore, to determine whether PI3K deficiency could alter HSC differentiation, we generated triple knockout (TKO) mice with deletion of all three class IA PI3Ks: P110α, P110β, and P110δ in hematopoietic cells. We previously demonstrated that, in HSCs, individual PI3K isoforms are dispensable for steady-state hematopoiesis but that P110α and P110δ play redundant roles during the HSC stress response (*14*–*16*). In our TKO model, we found that deletion of all three PI3K isoforms generated a phenotype resembling MDS, characterized by HSC expansion, impaired differentiation, multilineage dysplasia, and genomic instability. We found that one key mechanism responsible for this HSC differentiation defect is impaired autophagic degradation in TKO HSCs. We observed a similar autophagic degradation defect in patient MDS stem cells. Furthermore, we found that HSC differentiation can be improved in TKO mice through pharmacologic induction of autophagy. Therefore, we uncovered an essential role for class IA PI3K in HSC differentiation and in the regulation of autophagy in HSCs.

## RESULTS

**Fig. 1.**
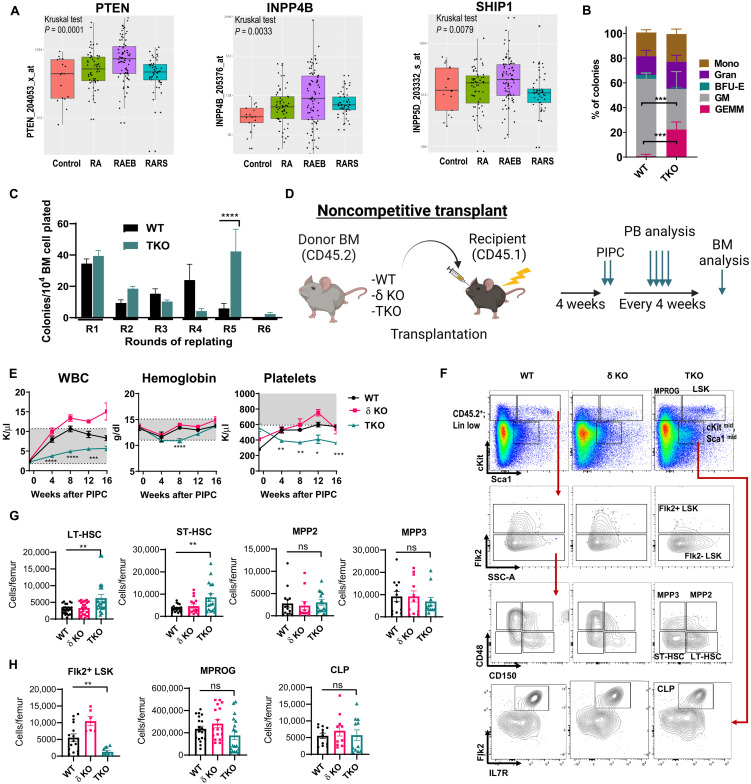
Class IA PI3K deletion leads to cell-autonomous pancytopenia and abnormal self-renewal. (**A**) Analysis of *PTEN*, *INPP4B*, and *SHIP1* expression in the MDS gene set GSE 19429 classified by French-American-British (FAB) subtype versus control healthy CD34^+^ cells. RARS, refractory anemia with ringed sideroblasts. (**B**) Relative frequencies of BM colonies plated in methylcellulose supplemented with myeloid growth factors (*N*_WT_ = 4, *N*_δKO_ = 4, and *N*_TKO_ = 4). BFU-E, burst-forming units-erythroid; GM, granulocyte-macrophage. (**C**) Quantitative analysis of WT and TKO BM serial replating assay round 1 through round 6 (R1 to R6) in methylcellulose (*N*_WT_ = 12 and *N*_TKO_ = 10). (**D**) Experimental design of noncompetitive BM transplantation (BMT; created with Biorender.com). (**E**) Longitudinal analysis of blood counts from WT, δ KO, and TKO BM recipients (*N*_WT_ = 7, *N*_δKO_ = 9, and *N*_TKO_ = 9). WBC, white blood cells. (**F**) Representative flow cytometry plots gated on the CD45.2^+^ lineage-low population from WT, δ KO, and TKO BM at 8 weeks after polyI-polyC (PIPC) from noncompetitive BM transplant mice. (**G** and **H**) Absolute numbers of donor-derived CD45.2^+^ cells per femur of (G) LT-HSCs, ST-HSCs, MPPs (MPP2 and MPP3), (H) Flk2^+^ LSK, myeloid progenitors (MPROG), and common lymphoid progenitors (CLP). Representative graphs from each experiment are shown. Each experiment was performed at least three times. WT;Mx1-Cre (WT) and p110δ KO (δ KO) mice were used as controls for TKO;Mx1-Cre mice (TKO). Immunophenotypic populations were defined as follows: LT-HSCs, Lin^−^cKit^+^Sca1^+^Flk2^−^CD48^−^CD150^+^; ST-HSCs, Lin^−^cKit^+^Sca1^+^Flk2^−^CD48^−^CD150^−^; MPP2, Lin^−^cKit^+^Sca1^+^Flk2^−^CD48^+^CD150^+^; MPP3, Lin^−^cKit^+^Sca1^+^Flk2^−^CD48^+^CD150^−^; MPROG, Lin^−^cKit^+^Sca1^−^; LSK, Lin^−^cKit^+^Sca1^+^; and CLP, Lin^−^cKit^mid^Sca1^mid^Flk2^+^IL7R^+^. Significance was determined using *t* test (B and C) or one-way analysis of variance (ANOVA) with Tukey’s multiple comparisons test (E, G, and H). *P ≤ 0.05, **P ≤ 0.01, ***P ≤ 0.001, and ****P ≤ 0.0001. ns, not significant.

Several studies have suggested that PI3K/AKT signaling is down-regulated in MDS patient samples (*11*, *12*). We examined the expression of PI3K pathway genes in the largest previously published expression dataset GSE19429 from MDS patient CD34^+^ cells relative to healthy CD34^+^ cells (*13*). We did observe significant decreases in *PIK3CA* expression with two of three probes and a trend toward decreased *PIK3CD* expression with one of two probes, but *PIK3CB* expression appeared unchanged (fig. S1, A to C). In addition, we observed a significant increase in the expression of the phosphatases *PTEN*, *INPP4B*, and *SHIP1* in MDS CD34^+^ cells compared to healthy CD34^+^ cells, particularly in the refractory anemia (RA) with excess blasts (RAEB) subset of high-risk MDS cases (Fig. 1A). Because up-regulation of each of these phosphatases can negatively regulate PI3K/AKT signaling, this would be predicted to result in decreased PI3K/AKT signaling in MDS stem cells. In addition, to better understand the possible correlation between *PTEN*, *INPP4B*, and *SHIP1* expression in MDS patient CD34^+^ cells, we performed Spearman rank correlation analysis, comparing all of the probes associated with *PTEN*, *INPP4B*, and *INPP5D* (*SHIP1*) in the MDS microarray dataset GSE 19429. Assessment of the expression levels of these phosphatases in all 183 patients with MDS, or in the 135 RA and RAEB patient subset, revealed a trend toward anticorrelation between the expression of *PTEN* and *INPP5D* (*SHIP1*), while *PTEN* and *INPP4B* expression appear to be positively correlated (fig. S1). This suggests that the PI3K pathway may be inactivated via up-regulation of different phosphatases in an even larger subset of patients with MDS. These data led us to hypothesize that PI3K/AKT inactivation could lead to impaired HSC function and promote MDS initiation.

### Genetic deletion of PI3K leads to pancytopenia and abnormal self-renewal

To determine whether PI3K inactivation can impair HSC function, we deleted all three class IA PI3K isoforms (P110α, P110β, and P110δ) in mouse hematopoietic cells. To do this, we generated conditional TKO mice: *Pik3ca^lox/lox^; Pik3cb^lox/lox^; Pik3cd^−/−^;Mx1-Cre,* with germline deletion of P110δ, and conditional deletion of P110α and P110β using the Cre-loxP system, where Cre expression is driven by the pan-hematopoietic *Mx1* promoter. After inducing excision by intraperitoneal injection with the synthetic double-stranded RNA polyI;polyC (pIpC), which activates Mx1-Cre, we harvested bone marrow (BM) cells and plated them at single-cell density in methylcellulose media with myeloid growth factors to examine their differentiation capacity. While total colony numbers were similar in all groups when compared to *WT;Mx1-Cre* (WT) controls (fig. S2A), TKO BM cells formed a higher proportion of immature granulocyte-erythroid-monocyte-megakaryocyte (GEMM) colonies, at the expense of more mature colonies (Fig. 1B). In addition, passaging the cells at the same single-cell density every 7 days showed that TKO BM cells have extended serial replating capacity for up to five passages (Fig. 1C). These data suggest that deletion of PI3K in BM cells alters their differentiation capacity and induces pathologic self-renewal. Mx1-Cre was previously shown to cause excision in nonhematopoietic tissues, such as the liver and BM stromal cells (*17*, *18*). Therefore, to avoid the potentially confounding effects of Mx1-Cre–mediated excision of PI3K isoforms in nonhematopoietic tissues and to focus on the effects of PI3K isoform deletion in hematopoietic cells over a longer observation period, we performed all remaining studies with BM transplantation (BMT) from 4- to 6-week-old donor mice.

To assess the self-renewal and differentiation properties of TKO BM cells in vivo, we performed noncompetitive BMT into lethally irradiated B6.SJL mice, followed by excision of P110α and P110β at 4 weeks after transplantation (Fig. 1D). To confirm inactivation of PI3K signaling, we measured AKT and MTOR phosphorylation in TKO hematopoietic stem and progenitor cells (HSPCs) by intracellular phospho-flow cytometry with ex vivo stem cell factor (SCF) stimulation. As expected, donor TKO HSPCs (LSK: Lin-Sca1^+^cKit^+^) had lower levels of AKT phosphorylation at Ser^473^ (fig. S2A) and of two downstream effectors of MTOR signaling, ribosomal protein S6 (Ser^235/236^) and 4EBP1 (Thr^37/46^; fig. S2, B and C). Longitudinal evaluation of the peripheral blood (PB) revealed cytopenias in TKO transplant recipients but not in control transplant recipients (WT or δKO littermate controls; Fig. 1E). Unexpectedly, BM flow cytometry analysis using previously defined markers (*19*) demonstrated an increase in the absolute number of donor-derived (CD45.2^+^) TKO long-term HSCs (LT-HSC) and short-term HSCs (ST-HSC; Fig. 1, F and G) but not of more mature populations (Fig. 1, F to H). We observed a significant decrease in the number of Flk2^+^ LSK cells, also known as lymphoid-primed multipotential progenitors (MPPs; Fig. 1, F and H) (*20*). Thus, we concluded that genetic inactivation of PI3K/AKT signaling in HSCs leads to pancytopenia and abnormal HSC expansion, similar to what is observed in patients with MDS.

**Fig. 2.**
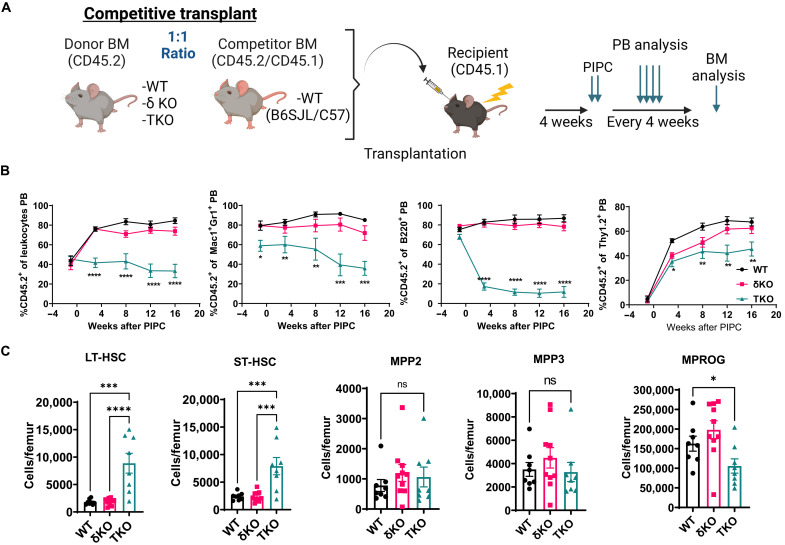
Class IA PI3K deletion leads to a competitive disadvantage in multilineage differentiation. (**A**) Experimental design of competitive BMT (created with Biorender.com). (**B**) Longitudinal analysis of total donor chimerism and myeloid and lymphoid donor chimerism in the PB (*N*_WT_ = 7, *N*_δKO_ = 9, and *N*_TKO_ = 9). (**C**) Absolute numbers of donor-derived CD45.2^+^ cells per femur at 16 weeks after PIPC of LT-HSCs, ST-HSCs, multipotent progenitors (MPP2 and MPP3), and MPROG. Representative graphs from one experiment are shown. The experiment was performed three times. WT;Mx1-Cre (WT) and p110δ KO (δKO) mice were used as controls for TKO;Mx1-Cre mice (TKO). Immunophenotypic populations were defined as follows: LT-HSCs, Lin^−^cKit^+^Sca1^+^Flk2^−^CD48^−^CD150^+^; ST-HSCs, Lin^−^cKit^+^Sca1^+^Flk2^−^CD48^−^CD150^−^; MPP2, Lin^−^cKit^+^Sca1^+^Flk2^−^CD48^+^CD150^+^; MPP3, Lin^−^cKit^+^Sca1^+^Flk2^−^CD48^+^CD150^−^; and MPROG, Lin^−^cKit^+^Sca1^−^. Significance was determined using one-way ANOVA with Tukey’s multiple comparisons test *P ≤ 0.05, **P ≤ 0.01, ***P ≤ 0.001, and ****P ≤ 0.0001.

To determine whether TKO BM can gain a competitive advantage over WT cells, we transplanted TKO BM cells in a 1:1 ratio with WT BM cells into lethally irradiated recipient mice, followed by pIpC treatment at 4 weeks after transplantation (Fig. 2A). TKO BM recipients exhibited a significant and persistent reduction in PB donor chimerism in both myeloid and lymphoid lineages (Fig. 2B). Notably, we again observed a significant increase in the absolute numbers of HSCs in the BM of TKO transplant recipients but not of more mature populations (Fig. 2C), with a decrease in the number of myeloid progenitors (MPROG), suggesting inefficient differentiation past the ST-HSC stage. Together, these data highlight the extensive redundancy between the class IA PI3K isoforms in HSCs, where deletion of P110α, β, and δ isoforms leads to perturbed self-renewal and decreased differentiation, resulting in ineffective hematopoiesis.

### Deletion of class IA PI3K leads to dysplastic changes in the BM

**Fig. 3.**
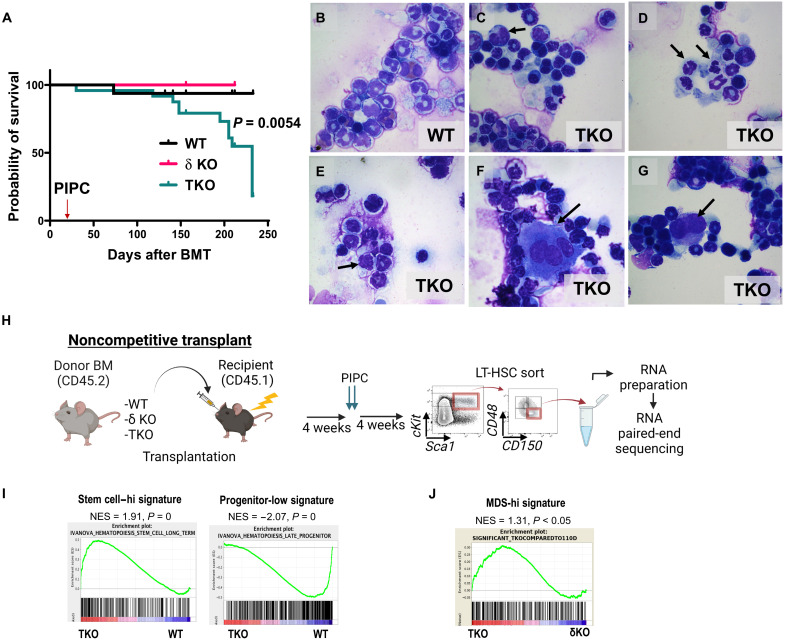
Class IA PI3K deletion in the BM leads to decreased survival, myelodysplasia, and gene expression changes associated with MDS. (**A**) Kaplan-Meier survival curve of noncompetitively transplanted animals (*N*_WT_ = 25, *N*_δKO_ = 14, and *N*_TKO_ = 27). Significance was determined using the log-rank (Mantel-Cox) test. (**B** to **G**) Photomicrographs of Wright-Giemsa–stained BM cytospins from a WT primary transplant recipient (control) and TKO primary transplant recipients at 8 weeks after transplantation (arrows point to dysplastic cells). (**H**) Experimental design for noncompetitive BMT and RNA sequencing sample preparation (created with Biorender.com). (**I**) GSEA of the TKO versus WT HSC signature with LT-HSC and progenitor signatures. NES, normalized enrichment score. (**J**) GSEA comparison between the TKO versus δKO HSC gene set and patient MDS versus healthy CD34^+^ cell signatures [GSE 19429; Pellagatti *et al.* (*13*)].

After noncompetitive BMT, TKO BM recipients had a median survival of 232 days, which is significantly shorter than that of WT or δKO littermate controls (Fig. 3A). While we observed normal trilineage differentiation in the BM of WT transplant recipients (Fig. 3B), BM cytospins from TKO transplant mice revealed multiple dysplastic changes in all three lineages (Fig. 3, C to G). An independent review by a hematopathologist revealed binucleated erythroid precursors (Fig. 3C), cells with irregular nuclear budding and irregular nucleation (Fig. 3D), neutrophils with hypersegmented nuclei (Fig. 3E), and megakaryocytes with multiple separated nuclei (Fig. 3F) or with small hyposegmented nuclei (Fig. 3G). These dysplastic features are similar to those observed in patients with MDS (*21*).

To determine the effects of PI3K isoform deletion on gene expression in HSCs, we performed bulk paired-end RNA sequencing on donor-derived TKO or control (δKO littermate or WT) sorted LT-HSCs from transplanted mice (Fig. 3H and table S1). Venn diagram analysis revealed many uniquely up-regulated and down-regulated genes between TKO and δKO HSCs, in addition to the differentially expressed genes between WT HSCs and HSCs from δKO littermate controls, which do not have a dysplastic phenotype (fig. S3A) (*16*). Gene set enrichment analysis (GSEA; MsigDB) (*22*) revealed that TKO LT-HSCs, compared with WT, have significant enrichment of both murine and human HSC signatures (*23*, *24*) and negative enrichment of late progenitor signatures (Fig. 3I and fig. S3B). To determine whether TKO HSCs have similar gene expression changes to those observed in human MDS CD34^+^ cells, we compared our TKO HSC gene expression signature to the MDS expression signature GSE 19429 (*13*). GSEA revealed enrichment of this MDS signature in our TKO HSC versus δKO HSC dataset (Fig. 3J).

**Fig. 4.**
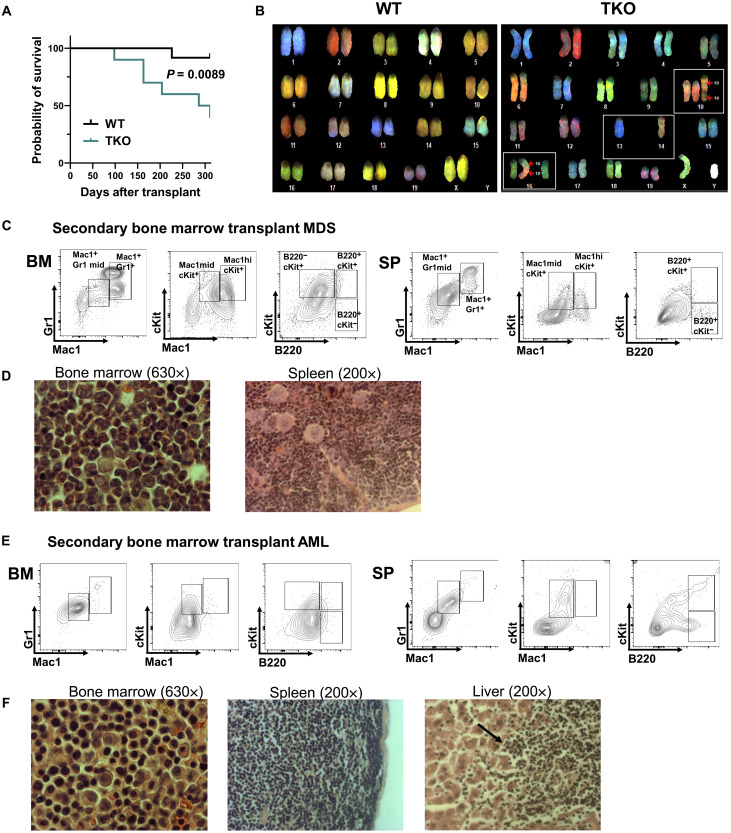
Serial transplantation of TKO BM cells promotes cytogenetic changes and progression to AML. (**A**) Kaplan-Meier survival curve of secondary BM transplant recipients. Significance was determined by the log-rank (Mantel-Cox) test (*N*_WT_ = 12 and *N*_TKO_ = 10). (**B**) Representative images of SKY chromosomal painting of donor-derived cKit^+^ WT or TKO cells from secondary transplant recipient mice. White boxes outline chromosomal abnormalities. *N* = 3 animals per genotype, with total cells analyzed per genotype: *N*_WT_ = 30 and *N*_TKO_ = 46. (**C**) Representative flow cytometry plots of BM and spleen (SP) and (**D**) photomicrographs of hematoxylin and eosin (H&E)–stained BM and spleen sections of a TKO secondary transplant recipient with MDS and extramedullary hematopoiesis. (**E**) Representative flow cytometry plots of BM and spleen (SP) and (**F**) photomicrographs of H&E-stained sections of the BM, spleen, and liver of a TKO secondary transplant recipient with AML.

To test whether the myelodysplastic changes in TKO transplant recipients can progress to more aggressive disease, we performed serial transplantation of TKO BM. We observed that, compared with WT control transplant recipients, TKO secondary transplant recipients had significantly shortened survival (Fig. 4A). Because MDS is often associated with cytogenetic abnormalities, we hypothesized that genomic instability could contribute to disease progression in our TKO BM transplant model. Spectral karyotyping (SKY) analysis on c-Kit^+^ BM cells from TKO secondary transplant recipients revealed multiple cytogenetic abnormalities, including translocations, monosomies, or trisomies (Fig. 4B). None of these changes were observed in WT secondary transplant control cells (Fig. 4B). Most secondary TKO transplant recipients also had dysplastic changes in the BM, with expansion of Mac1^+^Gr1^+^B220^−^cKit^+^ myeloid cells in the BM and spleen and extramedullary hematopoiesis in the spleen (Fig. 4, C and D). A minority (10%) of secondary TKO transplant recipients developed more aggressive disease resembling AML, with complete distortion of the spleen architecture and an accumulation of myeloblasts in the BM, spleen, and liver, which could also be observed with tertiary transplantation (Fig. 4, E and F, and fig. S4, A and B). Therefore, deletion of class IA PI3K isoforms causes a transplantable MDS-like phenotype, which can progress to AML. Together, these data reveal that our TKO model recapitulates several features of human MDS: impaired HSC differentiation, cytopenias, multilineage dysplasia, chromosomal abnormalities, and progression to AML in a subset of cases.

### Autophagy is dysregulated in TKO HSCs

To determine the mechanism for HSC expansion and impaired differentiation in TKO mice, we examined apoptosis and cell cycle progression by flow cytometry. Notably, the frequency of apoptotic cells (annexin V^+^PI^−^) was comparable between control and TKO cells (fig. S5A). In addition, there was no difference in the cell cycle profile between transplanted WT, δ KO, and TKO HSCs by flow cytometry for Hoechst 33342/Ki67 (fig. S5B).

Macroautophagy (henceforth referred to as autophagy) is a cellular recycling mechanism in which double-membraned vesicles, called autophagosomes, enclose intracellular material and fuse with lysosomes to degrade the engulfed cellular components (*25*). Autophagy plays an important role in the maintenance of quiescence and stemness in HSCs (*26*–*28*), and the PI3K pathway is a major conserved sensor for autophagy regulation. The PI3K/AKT downstream target MTOR has an inhibitory effect on autophagy, as its activation results in reduced autophagosome formation. Therefore, we hypothesized that dysregulated autophagy could lead to impaired HSC differentiation in TKO mice.

**Fig. 5.**
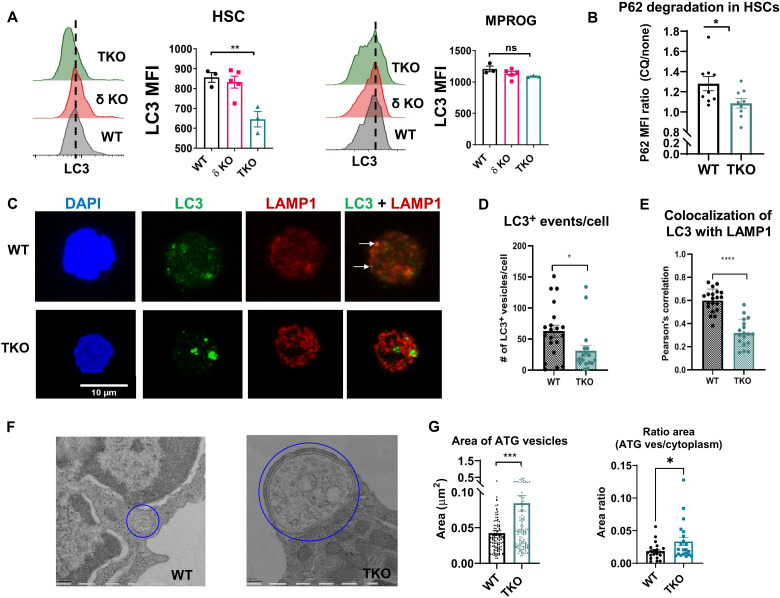
Loss of class IA PI3K leads to decreased autophagy in HSCs. (**A**) Representative LC3 flow cytometry analysis histograms and quantification of median fluorescent intensity (MFI) of LC3 in WT, δKO littermate control, and TKO HSCs and MPROG after serum and cytokine starvation (*N*_WT_ = 3, *N*_δKO_ = 5, and *N*_TKO_ = 3). (**B**) Quantification of P62 degradation in serum- and cytokine-starved WT and TKO HSCs with and without chloroquine (CQ) treatment, calculated as the ratio of P62 MFI treated with CQ/P62 MFI without CQ treatment (*N*_WT_ = 9 and *N*_TKO_ = 9). (**C**) Representative confocal images of sorted LT-HSCs stained with 4′,6-diamidino-2-phenylindole (DAPI; blue), anti-LC3 antibody (green), and anti-LAMP1 antibody (red). Colocalization of LC3 with LAMP1 appears yellow (see arrows). (**D**) Quantification of LC3^+^ events per cell and (**E**) colocalization events of LC3 with LAMP1 assessed by Pearson’s correlation (*N* = 20 cells per genotype from three independent samples). (**F**) Representative EM images of autophagic vesicles in sorted WT and TKO HSCs (*N* = 20 cells per genotype). (**G**) Quantification of the average area of autophagic vesicles (*N*_WT_ = 117 and *N*_TKO_ = 141) in WT versus TKO HSCs and of the average ratio of autophagic vesicles to cytoplasm per cell in WT and TKO HSCs (*N*_WT_ = 23 and *N*_TKO_ = 25). (A to H) Immunophenotypic populations were defined as follows: HSCs, Lin^−^cKit^+^Sca1^+^Flk2^−^CD48^−^; LT-HSC, Lin^−^Sca1^+^cKit^+^Flk2^−^CD48^−^CD150^+^; MPROG, Lin^−^cKit^+^Sca1^−^; and LSK, Lin^−^cKit^+^Sca1^+^. Significance was determined using one-way ANOVA with Tukey’s multiple comparisons test (A) or *t* test (B, D, and F) *P ≤ 0.05, **P ≤ 0.01, ***P ≤ 0.001, and ****P ≤ 0.0001. Representative data from individual experiments are shown. Each experiment was performed at least three times.

Consistent with this hypothesis, further analysis of our RNA sequencing data revealed negative enrichment of an autophagy gene signature in TKO HSCs and down-regulation of multiple genes in the autophagy pathway and network (fig. S5, C and D). We measured the levels of autophagy by intracellular flow cytometry using the autophagosomal marker microtubule-associated protein 1A/1B light chain 3B (LC3) protein (*29*) and the cargo protein marker p62 [also known as sequestosome 1 (SQSTM1)] (*30*), together with our cell surface panel for HSCs and progenitors. We found that, upon serum and cytokine starvation, LC3 staining was significantly decreased in TKO HSCs but not in MPROGs (Fig. 5A), suggesting an HSC-specific decrease in autophagy.

We hypothesized that the decrease in LC3 is due to a decrease in autophagic flux, with formation of fewer autophagosomes and less efficient cargo degradation. To examine autophagic flux in TKO cells, we assessed the degradation efficiency of the autophagy cargo protein P62 in starved cells with or without chloroquine, which blocks autophagosomal degradation by lysosomes. Compared with WT HSCs, TKO HSCs have an increase in steady-state levels of P62 and reduced degradation through autophagy, as demonstrated by the lower fold increase in P62 cellular content upon chloroquine treatment in these cells (Fig. 5B and fig. S5E). Therefore, our data reveal that loss of class IA PI3K in HSCs leads to reduced autophagic flux and defective cargo degradation.

To further understand the autophagy impairment in TKO HSCs and to assess the formation and degradation of autophagic vesicles, we performed immunostaining for LC3 in sorted serum-starved HSCs and analyzed it by confocal microscopy. Consistent with our LC3 flow cytometry findings, the confocal images showed that TKO LT-HSCs are able to induce autophagy and form autophagosomes, although we observed a decreased number of LC3^+^ events per cell, suggesting a decrease in the number of autophagic vesicles formed (Fig. 5, C and D). To examine the fusion of autophagosomes with lysosomes in the cell, we analyzed colocalization of LC3 with the endolysosomal marker lysosomal associated membrane protein 1 (LAMP1) (*31*, *32*), where colocalization events appear yellow (Fig. 5C). In TKO LT-HSCs compared with WT LT-HSCs, we observed significantly fewer colocalization events of LC3 with LAMP1 by Pearson’s correlation (Fig. 5E). This suggests that there is reduced induction of autophagy in TKO HSCs, along with fewer fusion events between autophagosomes and lysosomes. In further support of reduced clearance of autophagosomes by lysosomes in TKO HSCs, LC3^+^ autophagic vesicles appeared larger and brighter (Fig. 5C). Analysis of TKO and WT sorted HSCs by transmission electron microscopy confirmed that TKO autophagosomes are significantly larger than WT autophagosomes and occupy a significantly larger cytoplasmic area (Fig. 5, F and G). We noticed that the few autolysosomes (autophagosomes fused with lysosomes) that were detected in TKO cells were also larger and in a more immature state than those observed in WT cells, as they still contain partially degraded content (fig. S5F). Together, these findings demonstrate both inefficient autophagosome/lysosome fusion and decreased lysosomal degradation of autophagic cargo in TKO HSCs.

**Fig. 6.**
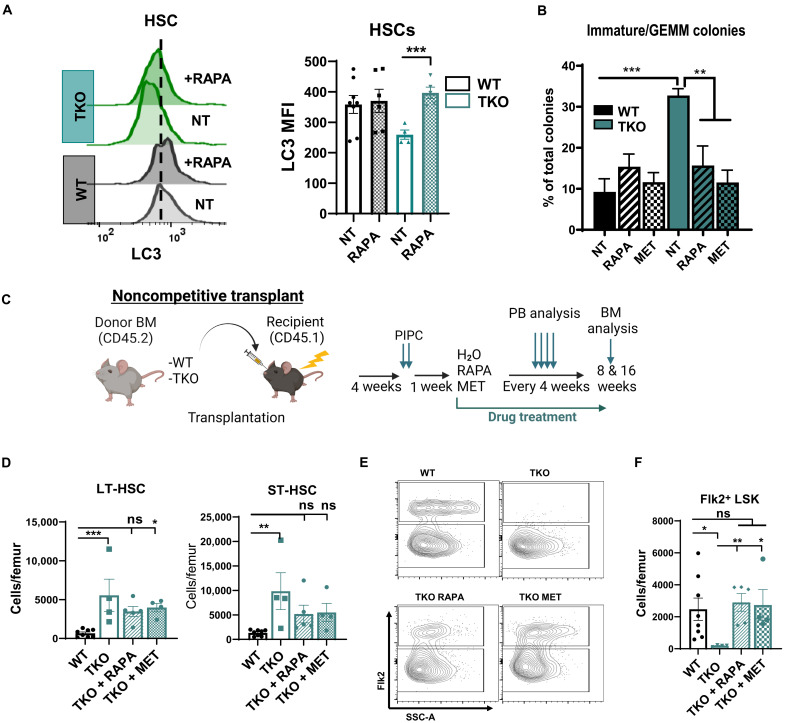
Pharmacologic up-regulation of autophagy in TKO HSCs improves differentiation and suppresses pathologic self-renewal. (**A**) Representative LC3II flow cytometry histograms and LC3II MFI quantification of WT and TKO HSCs on the right. After starvation, cells were either not treated (NT, PBS only) or treated with rapamycin (RAPA). (**B**) Quantification of GEMM colonies formed by WT and TKO BM cells in methylcellulose upon rapamycin (RAPA; 20 ng/ml) or metformin (MET; 50 mM) treatment (*N* = 4 per group). NT, no drug treatment (PBS only). (**C**) Experimental design of noncompetitive BMT with in vivo treatment with RAPA or MET (created with Biorender.com). At each time point, at least five animals per treatment group were analyzed. (**D**) Absolute numbers of donor-derived WT (*N* = 8) or TKO (*N* = 5 per group) LT-HSC and ST-HSC after 8 weeks of treatment. (**E**) Representative flow cytometry plots and (**F**) quantification of the number of donor-derived FLK2^+^ LSK cells in the BM after 8 weeks of in vivo treatment with rapamycin (RAPA; 15 mg/ml) or metformin (MET; 5 mg/ml). (A and D to F) Immunophenotypic populations were defined as follows: Flk2^+^LSK, Lin^−^Sca1^+^cKit^+^Flk2^+^; HSCs, Lin^−^Sca1^+^cKit^+^Flk2^−^CD48^−^; ST-HSCs, Lin^−^Sca1^+^cKit^+^Flk2^−^CD48^−^CD150^−^; and LT-HSCs, Lin^−^Sca1^+^cKit^+^Flk2^−^CD48^−^CD150^+^. Significance was determined using *t* test (D and F) or one-way ANOVA with Tukey’s multiple comparisons test (B). *P ≤ 0.05, **P ≤ 0.01, and ***P ≤ 0.001.

To determine whether the differentiation and self-renewal of TKO HSCs can be normalized by improving autophagic degradation, we treated TKO BM with two known autophagy-inducing drugs, rapamycin or metformin (*33*). Treatment of TKO BM cells with rapamycin ex vivo increased the levels of LC3 in both WT and TKO HSCs, supporting that induction of autophagy in TKO HSCs can be restored through this intervention (Fig. 6A). Notably, we found that treatment of TKO BM cells with either rapamycin or metformin in methylcellulose media led to a significant reduction in the frequency of immature GEMM colonies (Fig. 6B). To determine whether pharmacologic induction of autophagy could suppress pathologic HSC expansion and improve differentiation in vivo, we performed noncompetitive transplantation of TKO BM into lethally irradiated B6.SJL recipients and then treated transplanted mice with metformin or rapamycin in drinking water for 8 or 16 weeks, starting at 1 week after pIpC treatment, with plain water as a control (Fig. 6C). Both rapamycin and metformin were able to improve the differentiation of TKO HSCs at 8 weeks of drug treatment, as evidenced by a trend toward decrease in donor-derived LT-HSCs and ST-HSCs in TKO BM and significant recovery of the donor-derived Flk2^+^ LSK population (Fig. 6, D to F), which remained consistent at 16 weeks of treatment (fig. S6A). However, rapamycin or metformin treatment did not significantly affect LT- and ST-HSC populations in WT transplant recipients at the 8- or 16-week time points (fig. S6B). In addition, examination of BM cytospins from TKO mice after 8 weeks of drug administration revealed an improvement in myeloid differentiation with rapamycin or metformin treatment (fig. S6C). Consistent with the improvement in HSC differentiation with drug treatment, we also observed that metformin transiently improved platelet counts in TKO transplant mice (fig. S6D). As expected, 8 weeks of rapamycin or metformin treatment decreased P62 accumulation in WT BM and led to a significant decrease of P62 levels in the Flk2^+^ LSK population in TKO BM cells (fig. S6, E and F). We also observed a compensatory increase in phosphorylated extracellular signal–regulated kinase (pErk) in TKO HSCs at baseline and in the presence of rapamycin or metformin (fig. S6G), which could lead to residual MTORC1 signaling due to compensatory MTORC1 activation by pErk via tuberous sclerosis complex 2 inhibition (*34*).

### Human MDS stem cells have abnormal autophagic degradation

**Fig. 7.**
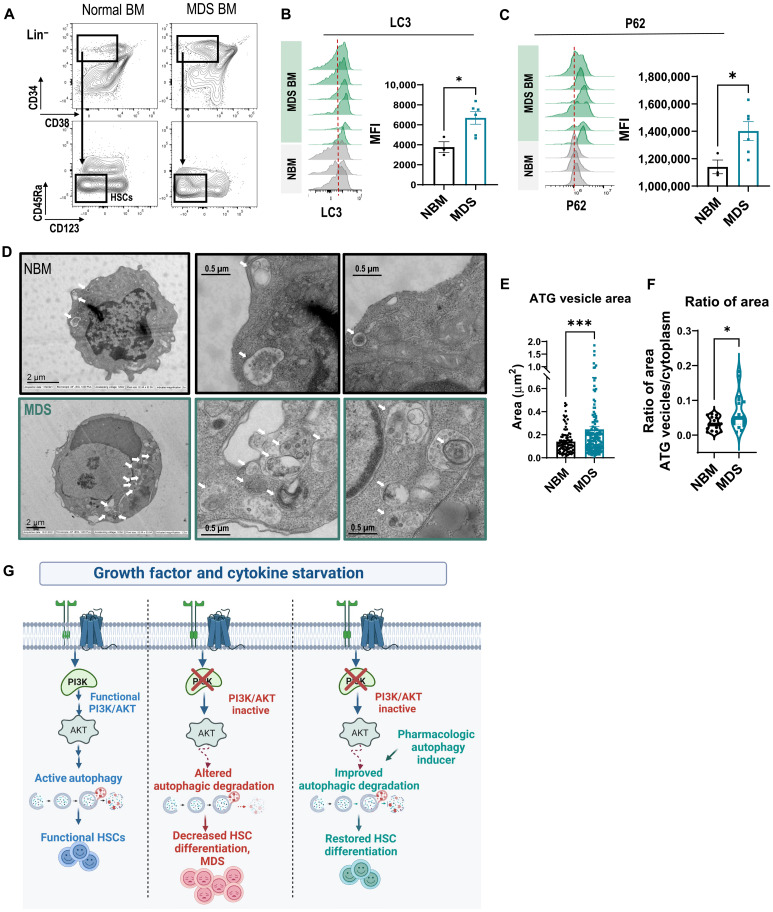
Human MDS stem cells have abnormal autophagic degradation. (**A**) Representative patient sample flow cytometry plots gated on Lin^−^ normal BM (NBM) and MDS BM. (**B** and **C**) Representative flow cytometry histograms and quantification of the MFI of (B) LC3 and (C) P62 (*N*_NBM_ = 3 and *N*_MDS_ = 6) in patient samples gated on the CD45Ra^−^CD123^−^ HSC population. (A to C) The experiment was performed three times with a total of NBM equal to 9 samples and 11 different MDS BM samples. (**D**) Representative electron microscopy images of autophagic vesicles from sorted CD34^+^ cells of NBM and MDS patient samples. (**E** and **F**) Quantification of the (E) area of autophagic vesicles (*N*_NBM_ = 92 and N_MDS_ = 143) and (F) ratio of total area of autophagic vesicles to area of the cytoplasm per cell in NBM versus MDS CD34^+^ cells (*N*_NBM_ = 15 and *N*_MDS_ = 18). (D to F) Analyzed images are from three different BM controls and three different patients with MDS. Significance was determined using Welsh’s *t* test *P ≤ 0.05 and ***P ≤ 0.001. (**G**) Current model: Under low cytokine and serum conditions, a functional PI3K/AKT pathway maintains autophagic degradation and supports functional HSCs. Inactivation of PI3K compromises autophagic degradation, leading to decreased HSC differentiation and MDS initiation. However, pharmacologic autophagy induction can bypass compromised PI3K/AKT activity, improving autophagic degradation and restoring HSC differentiation (image created with Biorender.com).

Given the autophagy defects that we observed in HSCs in our PI3K TKO mouse model, we hypothesized that autophagic degradation is also impaired in human MDS stem cells. To examine autophagy in MDS stem cells, we used our LC3 and p62 intracellular flow cytometry assay and adapted it with cell surface markers to identify human HSCs and progenitors. In the Lin^−^CD34^+^CD38^−^CD123^−^CD45Ra^−^ population, which is enriched for HSCs in both healthy BM and pre-MDS stem cells (*35*), we observed a significant increase in LC3 and P62 in pre-MDS stem cells, suggesting that these cells have perturbed autophagy and autophagosomal cargo accumulation (Fig. 7, A to C). Unexpectedly, we observed this pattern across multiple molecular and cytogenetic subtypes of MDS (table S2). Analysis of the Lin^+^CD33^+^ population, which contains MPROGs in control samples or blasts in MDS samples, showed no significant differences in the levels of LC3 and P62, suggesting highly variable levels of autophagy in more mature populations (fig. S7).

To visualize autophagic degradation in patient MDS cells, we performed transmission electron microscopy on sorted human CD34^+^ cells from healthy donor BM and MDS patient BM. In healthy BM samples, we could detect several small-sized autophagic vesicles in each cell plane (Fig. 7, D and E). As expected, in MDS patient samples, we observed an increase in the size of autophagic vesicles, which tend to cluster together and occupy a higher area of the cytoplasm (Fig. 7, D to F). This suggests that autophagic degradation is also impaired in human MDS stem cells, although autophagy was variable in more mature cell populations.

## DISCUSSION

In summary, our TKO mouse model recapitulates several key features of human MDS, including cytopenias, impaired HSC differentiation, genomic instability, multilineage dysplasia, and variable progression to AML. This work contributes a unique facet to the understanding of the role of PI3K/AKT signaling in the evolution of myeloid malignancies. While this pathway is frequently activated in AML blasts, the role of PI3K/AKT in MDS initiation is more complex and heterogeneous. Using our new PI3K TKO mouse model, we showed that genetic inactivation of PI3K/AKT impairs HSC differentiation, leading to pathologic self-renewal, myelodysplastic changes, and genomic instability.

In addition, we showed that PI3K inactivation can lead to impaired autophagic degradation in HSCs and that this is an important mechanism for impaired HSC differentiation. Autophagy can be used as a cellular protective mechanism from genomic instability, and defective autophagy has been associated with tumor initiation (*36*–*38*). Therefore, the increase in chromosomal abnormalities in TKO HSCs is consistent with the defective autophagy that we observed in TKO HSCs and the autophagy defects that we found in MDS patient HSCs. However, a direct causal link between impaired autophagy and genomic instability in MDS stem cells will require further investigation to establish.

This phenotype is markedly different from the effects of single PI3K isoform deletion or of P110α and P110δ compound deletion in HSCs, in which cases we did not observe any changes in HSC numbers or dysplasia (*15*, *16*). Therefore, these findings reveal the extensive redundancy between class IA PI3K isoforms in HSCs and underscore the importance of tight regulation of PI3K/AKT signaling to maintain the proper balance between HSC self-renewal and differentiation.

An important point to note is that the phenotypic analysis of *TKO;Mx1-Cre* mice was performed mostly in the transplantation setting to avoid potentially confounding effects of Mx1-Cre–mediated excision of PI3K isoforms in nonhematopoietic tissues, such as the liver and BM stromal cells, which precluded the long-term analysis of hematopoiesis in the primary *TKO;Mx1-Cre* mice. We did observe impaired myeloid differentiation and extended serial replating of primary BM cells in colony assays, suggesting that PI3K deletion is sufficient to cause some impairment of HSC/progenitor function without transplantation. However, it would be interesting in the future to determine whether PI3K deletion specifically in hematopoietic cells would be sufficient to lead to a similar MDS-like phenotype in vivo over time without the stress induced by transplantation using a different Cre model system.

While the role of class III PI3K in macroautophagy induction is well established, the connection between class I PI3K and autophagy is less well understood. We demonstrated that, upon loss of class IA PI3K signaling, which simulates the conditions of growth factor and cytokine starvation, autophagy plays a critical role in HSC self-renewal and differentiation. The most prominent known biological activity of class I PI3Ks is to generate PIP_3_, which leads to activation of AKT and MTOR signaling (*39*). Given that MTOR is a known negative regulator of autophagy, the prediction would be that class IA PI3Ks should also negatively regulate autophagy and that inactivation of PI3K should induce autophagy. Although we could detect autophagosome formation in PI3K TKO HSPCs, we demonstrated very inefficient autophagic degradation despite the decrease in AKT and MTOR signaling, suggesting an additional level of cross-talk between class I PI3K and autophagy.

The fact that the MTOR inhibitor rapamycin can still partially restore autophagy and improve the differentiation of TKO HSCs despite the baseline decrease in MTORC1 signaling that we observed in TKO cells is unexpected. This can partially be explained by the compensatory increase in pErk that we observed in TKO HSCs at baseline and in the presence of rapamycin or metformin, suggesting that MTORC1 is not completely abolished because of compensatory MTORC1 activation via the mitogen-activated protein kinase–ERK pathway (*34*). Furthermore, class I PI3Ks may regulate autophagy through both canonical AKT/MTOR-dependent and noncanonical MTOR-independent mechanisms. For example, alterations in autophagy flux have been demonstrated through changes in lysosomal activity or through the interactions between P110β and small guanosine triphosphatase Rab5 (*40*–*43*). It is also possible that this defect can be caused by a global change in the lysosomal program, as lysosomes have been shown to maintain quiescence of LT-HSCs through the endolysosomal trafficking of receptors (*42*). Thus, further studies will be needed to understand the mechanisms though which class IA PI3Ks regulate autophagic degradation.

We observed decreased expression of *PIK3CA* and a trend toward decrease in *PIK3CD* in the MDS expression dataset GSE 19429. In addition, we found that several phosphatases that are known to down-regulate PI3K signaling, *PTEN, SHIP1,* and INPP4B are significantly overexpressed in MDS patient CD34^+^ cells. Moreover, comparing the expression of these phosphatases, we observed a trend toward anticorrelation between the expression of PTEN and SHIP1, suggesting that at least one of these phosphatases is up-regulated in a large subset of patients with MDS. We anticipate that the elevated expression of these phosphatases in patients with MDS could lead to the functional inactivation of PI3K/AKT signaling in HSCs, which could contribute to impaired HSC differentiation and genomic instability via impaired autophagy. Consistent with this hypothesis, we found that human MDS stem cells have abnormal autophagy compared to healthy HSCs, similar to the autophagy defect that we observed in our PI3K TKO mouse HSCs.

However, we observed some variability in LC3 and P62 levels in MDS patient samples, which is expected, given the known molecular and phenotypic heterogeneity in MDS. We expected to observe variability in the autophagy phenotype in these MDS samples, especially because we purposely selected samples from patients representing multiple different MDS clinical subtypes and molecular and cytogenetic characteristics. To our surprise, we consistently observed an elevation in both P62 and LC3-II staining in the MDS stem cell compartment across MDS subtypes. More extensive future studies will be needed to explore the differences in autophagic degradation among the molecular and cytogenetic subtypes of MDS based on the international prognostic scoring system–molecular classification (*44*), which our current study is not powered to detect. In addition, in the future, it would be very interesting to assess the levels of phosphatase expression and AKT/MTOR activation in parallel to autophagy induction in each MDS patient sample. Overall, our data suggest that improved autophagic degradation can enhance the differentiation of PI3K-deficient HSCs, leading to an improvement in hematopoiesis (Fig. 7G). Future studies will be necessary to determine whether *PTEN, SHIP1*, and *INPP4B* up-regulation in stem cells is associated with specific clinical features in patients with MDS and whether these phosphatases could be used as biomarkers to select patients for specific therapeutic approaches.

Our finding that PI3K deletion promotes myelodysplasia may raise concerns regarding the use of PI3K inhibitors in the clinic. However, our TKO mouse model does not exactly represent the effects of the currently approved PI3K inhibitors. First, most of the currently approved PI3K inhibitors are selective for specific PI3K isoforms, or several isoforms, to avoid excessive toxicity. In our prior research, we have not observed a similar MDS phenotype with selective deletion of one or even two PI3K isoforms (*15*, *16*). Furthermore, all of the currently approved PI3K inhibitors are catalytic site inhibitors and do not affect total protein levels of the PI3K isoforms. However, in our TKO mouse model, there is complete deletion of the entire protein product of each of the three PI3K isoforms, so potential noncatalytic roles of the PI3K isoforms could also be affected. Therefore, we would not expect to observe the same risk of MDS in patients undergoing treatment with the currently available PI3K inhibitors. Furthermore, to our knowledge, there have been no reports of MDS or AML development that are attributed to the use of PI3K inhibitors in the clinic to date. However, we cannot rule out that prolonged use of multi-isoform PI3K inhibitors could increase the risk of MDS in patients, and it would be reasonable to monitor blood counts in these patients.

Our new mouse model of MDS would be useful in future studies to improve the understanding of MDS pathogenesis and to test therapeutic approaches for MDS. We demonstrated that the autophagy-inducing drugs rapamycin and metformin, both of which are clinically approved for other conditions, can suppress pathological self-renewal and improve hematopoietic differentiation of MDS cells in our TKO model. A large meta-analysis found that metformin use was associated with a decreased incidence of multiple cancer types and reduced the mortality of liver cancer and breast cancer (*45*). Furthermore, metformin has been reported to reduce DNA damage and improve hematopoiesis in a mouse model of Fanconi anemia (*46*). Therefore, there is a good preclinical rationale to test the effects of metformin in MDS. An ongoing clinical trial is investigating the preventative effects of metformin in patients with clonal cytopenias of undetermined significance and low-risk MDS (NCT04741945). In summary, our study provides strong evidence linking class IA PI3K and autophagy with the maintenance of HSC differentiation and suggests that further studies are needed to explore metformin and other autophagy inducers as a potential therapeutic approach for MDS.

## METHODS

### Human patient samples

Patient samples were obtained with written informed consent through the Montefiore Institutional Review Board biobank protocol no. 2005-536, in accordance with the Declaration of Helsinki. Donor BM samples were purchased from AllCells. All patient and BM donor characteristics are listed in table S2. The samples were defrosted in RPMI 1640, washed, and resuspended in RPMI 1640/FBS (fetal bovine serum) media (20% FBS, 1% penicillin-streptomycin, and 1% glutamine/GlutaMAX) and cultured for 1 hour. Cells were passed through a 30-μm cell strainer to remove dead cell clumps, washed with RPMI 1640, and resuspended in complete serum-free expansion medium media [with cytokines: hSCF (STEMCELL Technologies, 300-07) human thrombopoietin (STEMCELL Technologies, 300-18), human fms-related tyrosine kinase 3–Ligand (STEMCELL Technologies, 300-19) of 25 ng/ml and human interleukin-3 (hIL-3; STEMCELL Technologies, 200-03), hIL-6 (STEMCELL Technologies, 200-06) of 10 ng/ml]. Cells were incubated for 24 hours at 37°C and 5% CO_2_. To induce autophagy, the cells were washed with phosphate-buffered saline (PBS) three times and incubated in PBS for 2 hours at 37°C. To prepare for the flow analysis, cells were stained with Zombie NIR (1:2000) in PBS for 20 min at 4°C and washed with PBS. Cells were blocked on ice for 10 min with hCD16/CD32 in PBS. Cells were stained with cell surface antibody cocktail in PBS for 20 min on ice and washed with PBS. Cells were fixed with Cytofix/Cytoperm solution (BD Biosciences, BDB554714) 20 min on ice and washed with Perm/Wash buffer. Fixed cells were stained with cocktail of intracellular antibodies in Perm/Wash buffer for 20 min at room temperature, washed by Perm/Wash buffer, filtered by a 30-μm cell strainer, and analyzed by flow cytometry.

### MDS patient sample gene expression analysis

Analysis of *PTEN, INPP4B*, and *SHIP1* expression was performed on the MDS gene set GSE 19429, which was classified by French-American-British subtype versus control healthy CD34^+^ cells. The Spearman rank correlation was performed in pairwise combinations of probes associated with *PTEN*, *INPP4B*, and *INPP5D* (*SHIP1*) in the MDS microarray dataset GSE 19429. Expression of phosphatases was accessed in patient subsets of 183 patients with MDS and in the combined set of 135 RA and RAEB patients.

### Mice

Mice were maintained under pathogen-free conditions in a barrier facility in microisolator cages based on a protocol approved by the Institutional Animal Care and Use Committee at Albert Einstein College of Medicine (AECOM). *Pik3cd* germline KO mice (*47*) were provided as a gift by J. Ihle (St. Jude’s Children’s Hospital) as frozen embryos, rederived by the Boston Children’s Hospital Transgenic Facility, and backcrossed to the C57BL/6 strain for a total of 11 generations. To generate TKO mice, *Pik3ca*^lox/lox^;*Pik3cd^−/−^;*Mx1-Cre mice (*16*) were crossed with *Pik3cb*^lox/lox^ mice (*48*), resulting in progeny with the genotypes *Pik3ca*^lox/lox^;*Pik3cb*^lox/lox^;*Pik3cd^−/−^;*Mx1-Cre (TKO) and *Pik3ca*^lox/lox^;*Pik3cb*^lox/lox^;*Pik3cd^−/−^* (used as littermate controls). For *Pik3ca*^lox/lox^;*Pik3cb*^lox/lox^ excision, pIpC (Sigma-Aldrich) was dissolved in Hanks’ balanced salt solution, and 250 μg was injected intraperitoneally into 4- to 8-week-old mice three times on nonconsecutive days.

Experimental mice evenly included both males and females. PB was collected under isoflurane anesthesia by facial vein bleeding. Genotypes of each allele (*Pik3ca*, *Pik3cb*, and *Pik3cd*) were determined by polymerase chain reaction (PCR) using genomic DNA from tails as previously described (*47*–*49*). In addition, for each experiment, the excision status of exon 1 of *Pik3ca* and of exon 2 of *Pik3cb* was confirmed at least 2 weeks after pIpC injection using DNA from the BM or PB, as previously described (*48*, *49*). PB counts were evaluated on the Forcyte (Oxford Science) or Genesis (Oxford Science) blood analyzers.

### Colony formation unit assays and replating assays

Whole BM (WBM) cells were seeded at 10,000 cells per dish in methylcellulose (M3434, STEMCELL Technologies). The colonies were scored at 7 to 10 days after plating and replated with the same density into fresh M3434 methylcellulose media. For drug treatment, M3434 methylcellulose was supplemented with rapamycin (final concentration, 20 ng/ml; Santa Cruz Biotechnology, SC-3504) or metformin (final concentration, 50 μM; Enzo Life Sciences, 270-432-G005).

### BM transplantation

For all BMT, 6- to 8-week-old donor and recipient mice of both sexes were used unless stated otherwise. For noncompetitive BMT, 1 million WBM donor cells from C57BL/6 inbred mice (CD45.2^+^) were transplanted into lethally irradiated B6.SJL recipient (CD45.1^+^) mice. For competitive BMT, 500,000 CD45.2^+^ WBM donor CD45.2^+^ cells and 500,000 competitor cells from F1 progeny of B6.SJL and C57BL/6 (CD45.1^+^/CD45.2^+^) WBM cells were transplanted into lethally irradiated B6.SJL mice. For both noncompetitive and competitive BMT, donor mice were TKO: *Pik3ca*^lox/lox^;*Pik3cb*^lox/lox^;*Pik3cd^−/−^;*Mx1-Cre, δKO: *Pik3ca*^lox/lox^;*Pik3cb*^lox/lox^;*Pik3cd^−/−^* (littermate control), and WT: WT;Mx1-Cre (control). Recipient mice were given a single dose of irradiation (950 gray) at least 3 hours before retro-orbital transplantation, and recipient mice were given 0.9 ml of Baytril100 (100 mg/ml; Bayer) in 250 ml of drinking water for 3 weeks after transplantation. After confirmation of engraftment by PB counts, recipient mice were injected intraperitoneally with pIpC 250 μg twice, 48 hours apart. For serial BMT, noncompetitive transplant recipients were used as donors (20 to 28 weeks after transplantation); pooled BM from two or three animals was transplanted into lethally irradiated recipients.

### Drug treatment of mice with rapamycin or metformin

Metformin, rapamycin, and control water were administered to the transplanted mice for the duration of 8 or 16 weeks, starting at 1 week after pIpC treatment. Metformin (Enzo Life Sciences, catalog number 270-432-G005) was dissolved in drinking water (5 mg/ml) to achieve an average continuous level in the tissues and plasma of 32 μM (*50*). Rapamycin (Santa Cruz Biotechnology, catalog number SC-3504) was diluted in ethanol at a concentration of 15 mg/ml. This stock solution was further diluted 1:1000 in drinking water. Mice were expected to consume approximately 10% of their body mass in water daily, resulting in anticipated rapamycin consumption of 1.5 mg/kg per day (*51*, *52*). Metformin, rapamycin, and control water were administered to the transplanted mice for the duration of 8 or 16 weeks, starting at 1 week after pIpC treatment. The water was changed in the cages weekly.

### Flow cytometry

#### Live cells with cell surface markers

For flow cytometry analysis, BM from crushed or flushed bones was subjected to red blood cell (RBC) lysis, and BM cells were stained with a lineage cocktail of anti-mouse biotin-labeled lineage antibodies for 30 min at 4°C and anti-mouse fluorochrome-conjugated surface antibodies for 30 min at 4°C (table S3). PB cells were subjected to RBC lysis, blocked with CD16/CD32 block for 10 min on ice, and then stained with anti-mouse fluorochrome-conjugated surface antibodies for 30 min at 4°C (table S3). Flow cytometry analysis was performed on the BD FACS LSRII or Cytek Aurora. Analysis of all flow cytometry data was performed using FlowJo software (V9, V10). The absolute number of each population per femur was calculated on the basis of the number of WBM cells flushed from one femur and counted on the Countess instrument.

#### Fixed cells with intracellular markers

BM cells were harvested and stained with lineage and cell surface antibodies as described above (table S3), followed by mild fixation and permeabilization using the Cytofix and Cytoperm solutions from the BD Cytofix/Cytoperm Fixation/Permeabilization Solution Kit (BD Biosciences, BDB554714) according to the manufacturer’s protocol. Stained, fixed, and permeabilized cells were resuspended in Perm/Wash buffer containing antibody and stained with intracellular antibodies. Depending on the assay, fixed cells were subjected to one of the following intracellular staining protocols: (i) Cell cycle analysis. Permeabilized cells were incubated with Ki67-FITC (fluorescein isothiocyanate) overnight, followed by Hoechst 33342 (Invitrogen, H3570) staining at 25 μg/ml. (ii) Phospho-flow cytometry. After ex vivo stimulation, permeabilized cells were incubated with phospho-AKT (Ser^473^)–Alexa Fluor 647 (Cell Signaling Technology, 2337S) or pERK1(T202/Y204)–Alexa Fluor 488 (Cell Signaling Technology, 4374) at 1:20 dilution and phospho-S6 (Ser^235/236^)–Alexa Fluor 488 (Cell Signaling Technology, 4803S) or phospho-4E-BP1 (Thr^37/46^)–Alexa Fluor 647 (Cell Signaling Technology, 5123S) at 1:100 dilution. After intracellular staining, cells were washed with Perm/Wash buffer, resuspended in fresh Perm/Wash buffer, and analyzed by flow cytometry. For detailed antibody descriptions, see table S3. Flow cytometry was performed on the BD FACS LSRII or Cytek Aurora. Analysis of all flow cytometry data was performed using FlowJo software (V9 and V10). The absolute number of each population per femur was calculated on the basis of the number of WBM cells flushed from one femur and counted on the Countess instrument.

### Autophagy and cargo degradation analysis

For autophagy assessment, an intracellular flow cytometry protocol was adapted and modified from the FlowCellect Autophagy LC3 Antibody-based Assay Kit (Millipore, FCCH100171). After RBC lysis, freshly harvested BM cells were starved in PBS on ice for 2 hours to induce autophagy by serum and cytokine starvation. For the experiments where autophagy was induced by rapamycin, cells were treated for 2 hours by the Autophagy Reagent A (rapamycin containing) from the FlowCellect Autophagy LC3 Antibody-based Assay Kit (Millipore, FCCH100171), which have been diluted at 1:50 as per the manufacturer’s instructions. Following incubation, the cells were stained, fixed, and permeabilized using the Cytofix and Cytoperm solutions as described in the section above. For intracellular staining, cells were incubated in Perm/Wash buffer containing FlowCellect 20× optimized anti-LC3/FITC antibody or LC3A/B-AF488 (Cell Signaling Technology, 13082S) and anti-SQSTM1/p62 antibody Alexa Fluor 647 (ab194721) at 1:400 and incubated for 30 min at room temperature. Cells were washed with Perm/Wash buffer to remove residual unbound antibody and resuspended in fresh Perm/Wash buffer followed by flow cytometry analysis on the Cytek Aurora. Analysis of all flow cytometry data was performed using FlowJo software. For detailed antibody description, see table S3.

### RNA sequencing analysis

One million WBM cells from TKO, δ KO (littermate control), and WT (control) donor mice were transplanted into lethally irradiated 6- to 8-week-old B6.SJL recipient mice (*N* = 7 per group). After confirmation of engraftment by PB counts, recipient mice were injected intraperitoneally with pIpC 250 μg twice, 48 hours apart. Excision status of *Pik3ca* and *Pik3cb* was confirmed by PCR on BM DNA as described above. At 4 weeks after pIpC, BM cells were harvested from femurs, tibiae, ilia, and vertebrae by gentle crushing in RPMI 1640 (Life Technologies). Low-density BM mononuclear cells were isolated by density gradient centrifugation using Ficoll Histopaque 1083 (Sigma-Aldrich, 10831), pooled from all seven recipients in each group, and stained with anti-mouse biotin-labeled lineage antibodies for 30 min at 4°C. The lineage-stained cells were incubated with Biotin Binder Dynabeads (Thermo Fisher Scientific, 11047), followed by magnetic depletion according to the manufacturer’s protocol. Lineage-negative cells were stained with a panel of fluorochrome-conjugated monoclonal anti-mouse cell surface antibodies (table S3). The donor-derived LT-HSC cell populations were sorted according to the gating strategy (Fig. 3H) using MoFlo Astrios Cell Sorter (Beckman Coulter). LT-HSC cells were directly sorted into 50 μl of RNA extraction buffer, and RNA was prepared using the ARCTURUS PicoPure RNA Isolation Kit (Applied Biosystems, 12204-01) according to the manufacturer’s protocol and stored at −80°C. Frozen samples were shipped to the Beijing Genomics Institute for library preparation and sequencing. For transcriptome sequencing, cDNA was amplified from total RNA using the Ovation RNA-Seq System V2. Sequencing was performed using the NGS platform llumina-HiSeq2000 through collaboration with Beijing Genomics Institute (BGI). Raw fastq files were downloaded from BGI and subjected to analysis. Flanking adapter sequences were removed with Trim Galore (v.0.3.7; www.bioinformatics.babraham.ac.uk/projects/trim:galore/), and sequence quality was assessed using fastqc (v.0.11.4; www.bioinformatics.babraham.ac.uk/projects/fastqc/). Adapter-trimmed fastq files were aligned to the mouse mm10 genome, and read counts per gene were determined using the splice-aware aligner STAR (v2.5.1b) (*53*). Differential gene expression was assessed with R package DESeq2 (http://bioconductor.org/packages/release/bioc/html/DESeq2.html). Data were analyzed using GSEA using MSigDB software from Broad Institute (http://software.broadinstitute.org/gsea/index.jsp). Venn diagrams were constructed using the Venn diagram tool from Bioinformatics and Evolutionary Genomics (Ghent University; http://bioinformatics.psb.ugent.be/webtools/Venn/).

### Cytogenetics

At 22 weeks after transplantation, BM cells from secondary BMT mice were harvested from femurs, tibiae, pelvis, and vertebrae by gentle crushing in RPMI 1640 (Life Technologies). BM cKit^+^CD45.2^+^ cells were sorted using the MoFlo Astrios Cell Sorter. Cells (0.5 × 10^6^) were resuspended in 1 ml of BMT media: RPMI 1640, 10% FBS, 1% penicillin-streptomycin, recombinant mouse IL-3 (10 μg/ml; R&D Systems, #403-ML), 50 μl of recombinant mouse SCF (10 μg/ml; R&D Systems, #1832-01), 50 μl of recombinant mouse IL-6 (10 μg/ml; PeproTech, #200-06) and cultured at 37°C and 5% CO_2_ for 18 hours. Clocemid (0.01 μg/ml) was added to the cell suspension and incubated at 37°C overnight. The cells were washed with PBS and treated with hypotonic solution (0.075 M KCl) for 23 min, followed by fixation with methyl alcohol/glacial acetic acid (3:1). The cells were washed with 10 ml of freshly prepared fixative and centrifuged for 5 min at 1200 rpm. Resuspended cells in fixative were dropped on slides in Thermotron and imaged.

### Histology on BM, spleen, and liver

Wright-Giemsa staining of PB smears and cytospins was performed using the Hema 3 system (Fisher) as per the manufacturer’s protocol. Harvested murine tissues were fixed in 10% buffered formalin (Thermo Fisher Scientific, SF1004). At the Einstein Histopathology Core Facility, samples were embedded in paraffin, cut into thin slices, and mounted on a slide. Mounted tissues were stained with hematoxylin and eosin (H&E) stain and imaged.

### Autophagy immunofluorescence staining

Freshly harvested sorted LT-HSCs were starved in PBS for 2 hours and then fixed onto RetroNectin (Clontech)–coated slides. Cells were fixed with 4% paraformaldehyde in PBS, permeabilized with 0.25% Triton X-100 in PBS, and blocked with 1% bovine serum albumin in PBS. The cells were incubated with conjugated antibodies LC3A/B–Alexa Fluor 488 (Cell Signaling Technology, #13082) and LAMP1–Alexa Fluor 546 (Santa Cruz Biotechnology, SC-20011 AF546) in 4°C overnight and then washed with PBS. The nuclei were stained with 4′,6-diamidino-2-phenylindole VECTASHIELD mounting media (Vector Laboratories, H-1200) before imaging.

### Microscope image acquisition

Histology images were acquired using the Olympus BX43 microscope with a digital camera, using 20× and 63× oil immersion objective. Image acquisition was performed using Infinity software.

Immunofluorescence images were acquired using the Leica SP8 Confocal Microscope using a 63× oil immersion objective (working distance = 0.14). Image acquisition was performed using LASX software.

### Electron microscopy

LT-HSCs or human CD34^+^ cells were sorted in PBS, fixed in 2% paraformaldehyde and 2.5% glutaraldehyde in 0.1 M sodium cacodylate buffer, and then mixed with 1% osmium tetroxide in 0.1 M sodium cacodylate buffer after fixation. The cells were bloc-stained with 2% uranyl acetate (aq), dehydrated in a graded series of ethanol, and embedded in LX112 resin (LADD Research Industries, Burlington, VT) in Eppendorf tubes. Ultrathin sections were cut on a Leica Ultracut UC7 and stained with uranyl acetate, followed by lead citrate. Obtained sections were viewed and imaged on a JEOL 1200EX transmission electron microscope at 80 kV.

### Reagents

Please refer to table S3 for the list of flow cytometry antibodies and other antibodies used. All other reagents are listed in the respective Method sections.

### Study approval

All mouse breeding and animal experiments were approved by the Institutional Animal Care and Use Committee under protocol nos. 20170205, 20170206, 00001165, and 00001181.

### Statistics

GraphPad Prism 7 and 8 were used for all statistical analyses. For the comparison of two experimental groups, the unpaired two-tailed Student’s *t* test was used. For the comparison of greater than two groups, the analysis of variance (ANOVA) test was used with the Tukey’s multiple comparisons test, unless otherwise indicated. For survival analysis, log-rank (Mantel-Cox) test analysis was used. The specific statistical test used for each specific experiment is indicated in each figure legend. In all graphs, error bars indicate ±SEM. A *P* value of <0.05 was considered significant.
