# Supplementary material for: PI3-kinase deletion promotes myelodysplasia by dysregulating autophagy in hematopoietic stem cells
Source: Sci Adv. 2023 Feb 22;9(8):eade8222. doi: 10.1126/sciadv.ade8222 (PMC9946350; doi:10.1126/sciadv.ade8222)
Supplement: Supplementary file 1 — Figs. S1 to S7 Legends for tables S1 to S3 [file sciadv.ade8222_sm.pdf]

Science Advances
AAAS

Supplementary Materials for

PI3-kinase deletion promotes myelodysplasia by dysregulating autophagy in hematopoietic stem cells

Kristina Ames *et al.*

Corresponding author: Kira Gritsman, kira.gritsman@einsteinmed.edu

Sci. Adv. **9**, eade8222 (2023)
DOI: 10.1126/sciadv.ade8222

The PDF file includes:

Figs. S1 to S7
Legends for tables S1 to S3

Other Supplementary Material for this manuscript includes the following:

Tables S1 to S3

Supplementary Data

Supplementary Table S1:GSEA results

Supplementary Table S2: MDS patient samples and controls

Supplementary Table S3: Antibodies used in the study

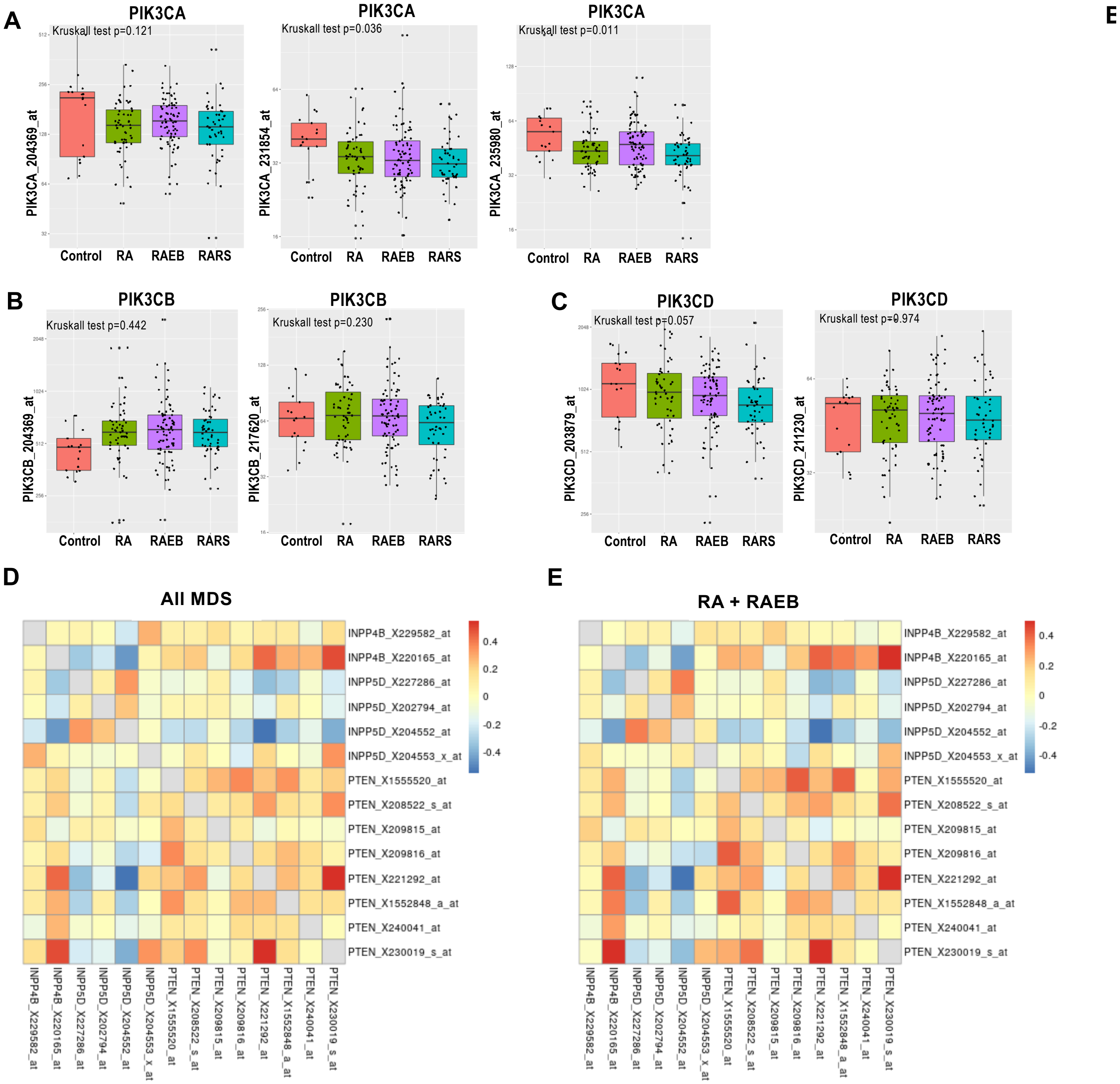

Fig. S1. Correlation between PI3KCA, PI3KCB, PI3KCD, PTEN, INPP5D, and INPP4B expression in MDS patients. (A-C) Analysis of *PI3KCA, PI3KCB, PI3KCD* expression in the MDS gene set GSE 19429 classified by French-American-British (FAB) subtype vs control healthy CD34⁺ cells. RA= refractory anemia, RAEB= refractory anemia with excess blasts, RARS= refractory anemia with ringed sideroblasts **(D,E)** Heat maps representing Spearmann rank correlation analysis comparing all the probes associated with *PTEN, INPP4B*, and *INPP5D* (*SHIP1*) in the MDS microarray dataset GSE 19429. Expression of phosphatases was examined in **(D)** 183 total MDS patients and in **(E)** the 135 patient RA and RAEB patient subset.

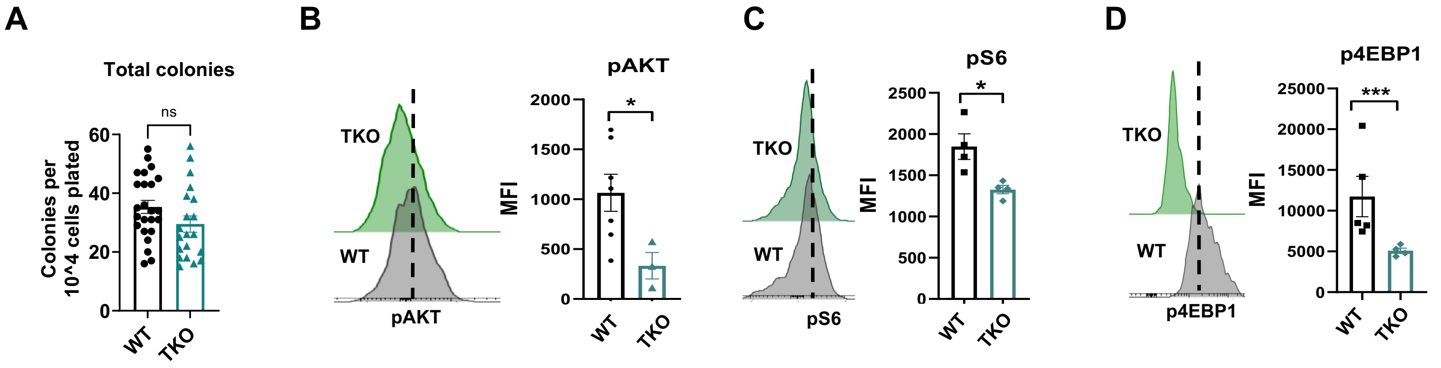

Fig. S2. Class IA PI3K deletion decreases AKT/MTOR signaling in hematopoietic stem and progenitor cells. (A) Total colony counts of TKO and WT bone marrow cells plated in M3434 methylcellulose media **(B-D)** Representative phospho-flow cytometry histograms and quantification of the median fluorescent intensity (MFI) of **(B)** pAKT (Ser 473)(N_{WT}=7, N_{TKO}=3), **(C)** pS6 (Ser235/236) (N_{WT}=4, N_{TKO}=4) and **(D)** p4EBP1 (Thr37/46) (N_{WT}=5, N_{TKO}=4) signal in the non-competitive transplant donor derived LSK population at 16 weeks post-PIPC. Cells were stimulated *ex vivo* for 5 minutes with SCF. **(B-D)** Immunophenotypic populations were defined as LSK: $Lin^-Sca1^+cKit^+$. Representative graphs of each experiment are shown. Each experiment was performed at least 3 times. WT;Mx1-Cre (WT) and p110δ KO (δ KO) mice were used as controls for TKO;Mx1-Cre mice (TKO). Significance was determined using the t-test **(A-D)** *P≤0.05, ***P ≤ 0.001, ns - no significance.

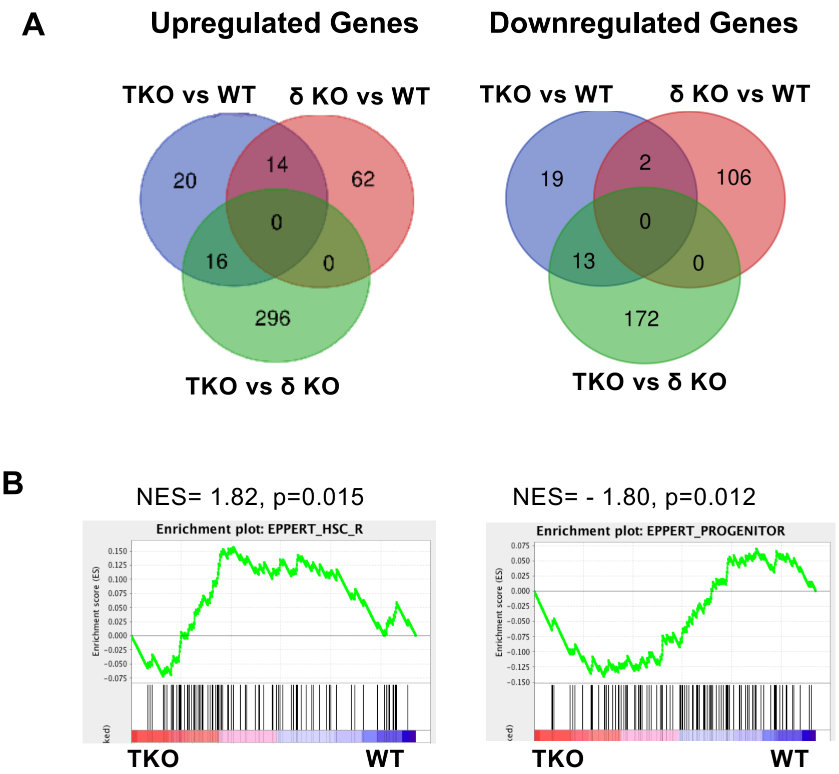

Supplementary Fig. S3. Class I PI3K deletion leads to dysregulated gene expression in HSCs. (A) Venn diagrams detailing shared and distinct gene expression changes among TKO, δKO and WT LT-HSCs. Genes were selected based on >2 fold change and adjusted p<0.05. **(B)** GSEA of the TKO vs. WT HSC signature with human HSC and progenitor gene sets (GSE30377; Eppert K., et al. *Nature Medicine*, 2011).

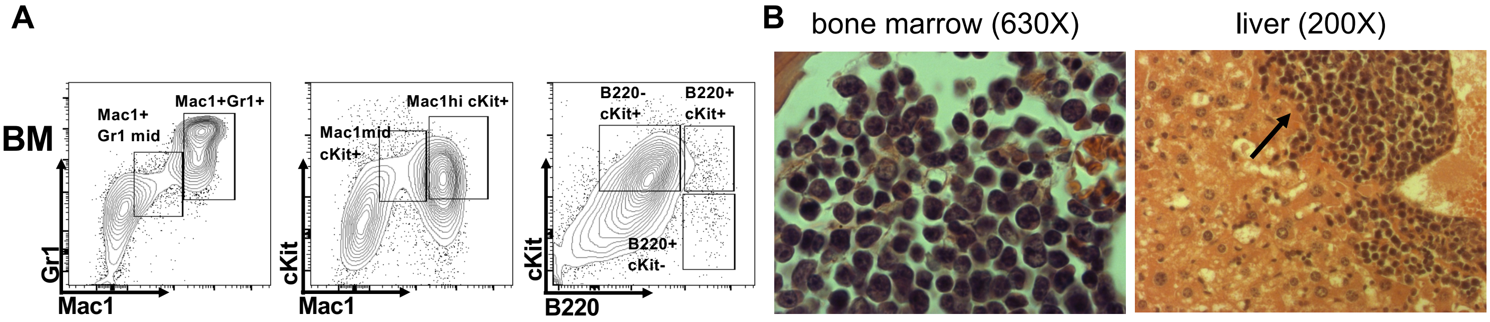

Supplementary Fig. S4. Serial transplantation of TKO bone marrow cells promotes progression to AML. (A) Representative flow cytometry plots of bone marrow (BM) and **(B)** photomicrographs of H&E-stained sections of the bone marrow and liver of a TKO tertiary bone marrow transplant recipient with AML.

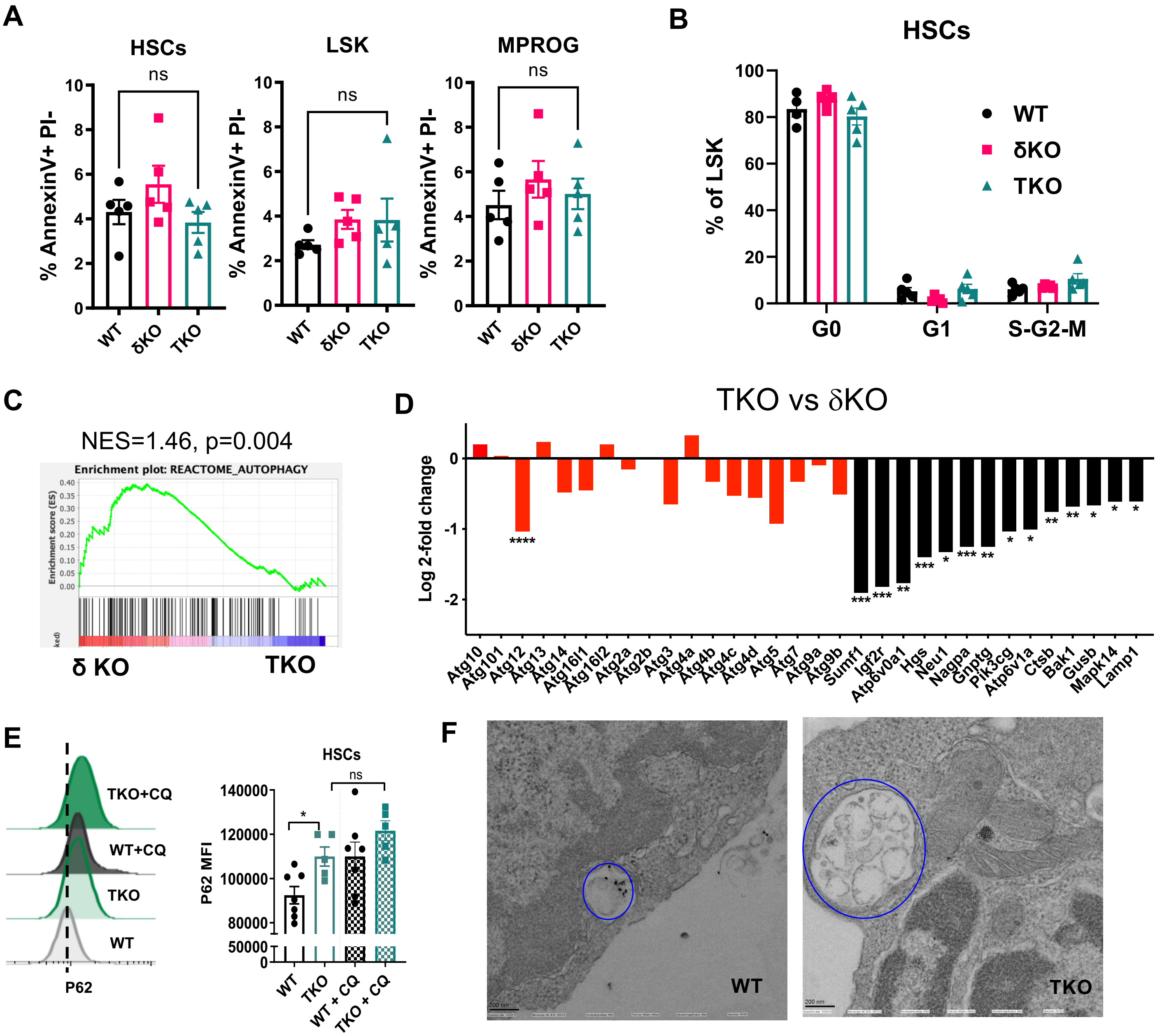

Supplementary Fig. S5. Class IA PI3K deletion in HSCs alters autophagy. (A) Quantification of apoptotic cells in the HSC, LSK and MPROG populations by Annexin V and propidium iodide (PI) staining (N_{WT}=5, $N_{\delta KO}$=4, N_{TKO}=5). **(B)** Quantification of the G0, G1, and S-G2-M cell cycle phases of donor LT-HSC at 8 weeks post-pIpC in non-competitive bone marrow transplant recipients (N_{WT}=4, $N_{\delta KO}$=5, N_{TKO}=5. **(C)** GSEA plot of the δ KO vs TKO LT-HSC gene set with the REACTOME_AUTOPHAGY gene set from MSigDB. **(D)** Comparison of the expression of individual manually curated autophagy genes (in red) and autophagy related genes (in black) in our RNA seq gene set between TKO and δ KO LT-HSCs. **(E)** Representative flow cytometry histograms and quantification of median fluorescent intensity (MFI) of P62 in serum- and cytokine-starved HSCs with and without chloroquine (CQ) treatment (N_{WT}=7, N_{TKO}=5). **(A,B, E)** Experiment was performed at least 3 times. **(F)** Representative electron microscopy (EM) images of autophagic vesicles in sorted WT and TKO HSCs (N= 20 cells per genotype). **(A-F)** Immunophenotypic populations were defined as follows: MPROG: $Lin^-Sca1^-cKit^+$, LSK: $Lin^-Sca1^+cKit^+$, HSCs: $Lin^-Sca1^+cKit^+Flk2^-CD48^-$. Significance was determined using the one-way ANOVA test with Tukey's multiple comparison's test. *P≤0.05, **P ≤ 0.01, ***P ≤ 0.001, ****P ≤ 0.0001.

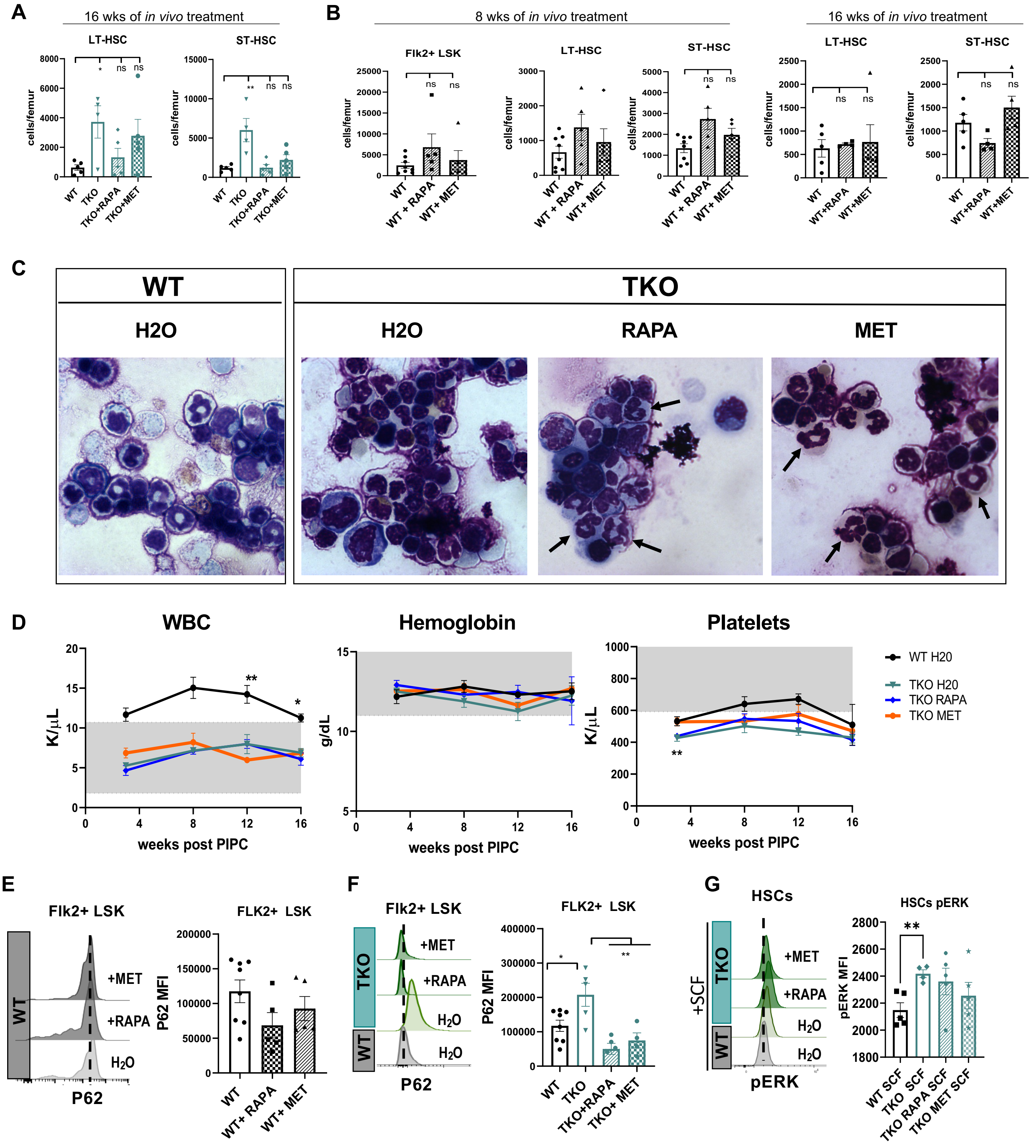

Supplementary Fig. S6. *In vivo* treatment with rapamycin or metformin does not affect WT HSCs but decreases pathologic expansion of HSCs in TKO bone marrow. (A) Quantification of donor-derived WT vs. TKO LT-HSC and ST-HSC cells after 16 weeks of *in vivo* treatment with rapamycin (RAPA) or metformin (MET) **(B)** Quantification of donor-derived WT Flk2+ LSK, LT-HSC and ST-HSC cells after 8 or 16 weeks of *in vivo* treatment with rapamycin (RAPA) or metformin (MET). **(C)** Photomicrographs of Wright-Giemsa-stained bone marrow cytospins from WT (control) and TKO transplant recipients after 8 weeks of *in vivo* treatment with rapamycin (RAPA) or metformin (MET). Images taken at 200x magnification. **(D)** Serial blood counts of non-competitive WT vs TKO transplant recipients during *in vivo* treatment with RAPA or MET **(E, F)** Representative flow cytometry histograms and quantification of median fluorescent intensity of P62 in starved **(E)** WT and **(F)** TKO Flk2+ LSK after 8-weeks of *in vivo* treatment with RAPA or MET. **(G)** Representative phospho-flow cytometry histograms and quantification of the median fluorescent intensity (MFI) of pERK (T202/Y204) (N_{WT}=5, N_{TKO}=4, $N_{TKO\ RAPA}$=5, $N_{TKO\ MET}$=5) signal after 16-weeks of *in vivo* treatment with RAPA or MET. Cells were stimulated *ex vivo* for 5 minutes with SCF. Immunophenotypic populations were defined as follows: Flk2$^+$LSK: Lin$^-$Sca1$^+$cKit$^+$Flk2$^+$, ST-HSCs: Lin$^-$Sca1$^+$cKit$^+$Flk2$^-$CD48$^-$CD150$^-$, LT-HSCs: Lin$^-$Sca1$^+$cKit$^+$Flk2$^-$CD48$^-$CD150$^+$, HSCs: Lin$^-$Sca1$^+$cKit$^+$Flk2$^-$CD48$^-$. Significance was determined using the one-way ANOVA test with the Tukey's multiple comparison's test. *P≤0.05, **P ≤ 0.01

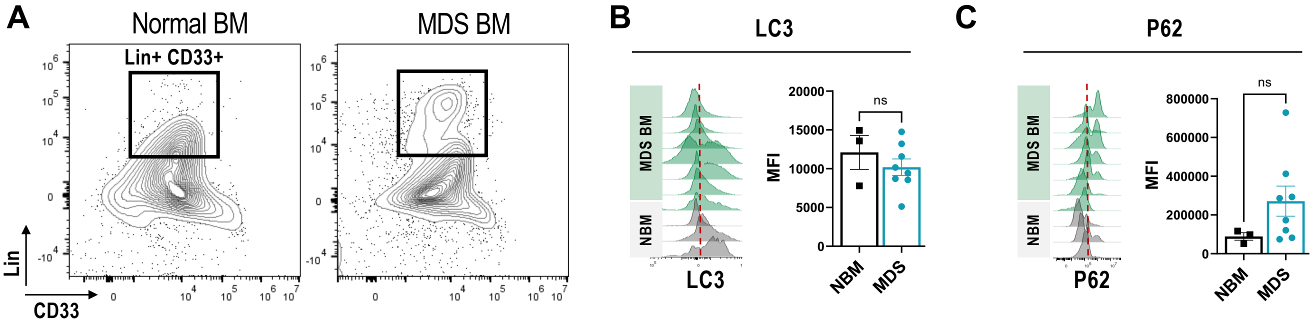

Supplementary Fig. S7. Myeloid blasts and myeloid progenitors have variable autophagic degradation. (A) Representative patient samples flow cytometry plots gated on Lin+ CD33+ cells in normal bone marrow (NBM) and MDS bone marrow. **(B,C)** Representative flow cytometry histograms and quantification of the median fluorescent intensity (MFI) of **(B)** LC3, **(C)** P62 (N_{NBM}=3, N_{MDS}=6) in patient samples gated on Lin+CD33+ cells population. Experiment was performed 3 times with total of NBM=9 samples and 11 different MDS BM samples. Significance was determined using the t-test *P≤0.05, **P ≤ 0.01, ***P ≤ 0.001, ****P ≤ 0.0001.
